# Gender Differences in the Association Between Obesity Indices and Chronic Kidney Disease Among Middle-Aged and Elderly Taiwanese Population: A Community-Based Cross-Sectional Study

**DOI:** 10.3389/fendo.2021.737586

**Published:** 2021-12-13

**Authors:** I-Ju Chen, Le-Tien Hsu, Mei-Chun Lu, Ying-Jen Chen, Meng-Ting Tsou, Jau-Yuan Chen

**Affiliations:** ^1^ Department of Family Medicine, Chang Gung Memorial Hospital, Taoyuan City, Taiwan; ^2^ Department of Gynecology and Obstetrics, Chang Gung Memorial Hospital, Taoyuan City, Taiwan; ^3^ Division of General Internal Medicine and Geriatrics, Department of Internal Medicine, Chang Gung Memorial Hospital, Taoyuan City, Taiwan; ^4^ Department of Family Medicine and Occupation Medicine, MacKay Memorial Hospital, Taipei City, Taiwan; ^5^ Department of Nursing, and Management, MacKay Junior College of Medicine, New Taipei City, Taiwan; ^6^ College of Medicine, Chang Gung University, Taoyuan City, Taiwan

**Keywords:** obesity index, chronic kidney disease, middle-aged and elderly, gender difference, visceral adiposity index

## Abstract

**Background:**

Traditional risk factors for chronic kidney disease (CKD) include diabetes mellitus (DM), hypertension (HTN), and metabolic syndrome, which are health conditions related to obesity. We aimed to investigate which of the three obesity indices has the strongest association with CKD and to explore whether there are gender differences in these relationships in the middle-aged and elderly Taiwanese population.

**Methods:**

This was a cross-sectional, community-based study. It included 400 residents (141 males and 259 females, age 50–90 years) residing in a community in northern Taiwan. Each participant was asked to fill a questionnaire that collected personal information, medical history, medication use, and anthropometric measurements. The laboratory data were obtained by testing the blood and urine samples. The baseline characteristics were compared, and the obesity indices included body mass index (BMI), waist circumference (WC), and visceral adiposity index (VAI). CKD was defined as the presence of renal dysfunction (urine albumin-creatinine ratio ≥ 30 mg/g) or estimated glomerular filtration rate (eGFR) < 60 mL/min/1.73m^2^. We used a multiple logistic regression model to evaluate the association between each obesity index and CKD for both genders. Further, we used the area under the receiver operating characteristic (ROC) curve (AUC) to examine the best obesity indices to predict CKD in different genders.

**Results:**

The average age of the subjects was 64.47 ± 8.45 years, and men were significantly older. CKD was found in 31 (22.0%) males and 50 (19.3%) females. In men, there was no significant difference between the CKD and non-CKD groups among the three obesity indices. However, in women, only VAI was significantly higher in subjects with CKD (1.9 [1.1, 3.4]) than in subjects without CKD (1.5 [1.0, 2.2]) (p-value = 0.03). The multivariate logistic regression revealed that even after adjusting for possible confounding factors, VAI was found to be an independent risk factor for CKD in women (OR: 1.32, 95% CI: 1.04-1.69, p = 0.02), but not in men (OR: 1.20, 95% CI: 0.85-1.69, p = 0.30). The AUC of VAI had a significant ability to predict CKD in women but not in men.

**Conclusion:**

Our results showed that among the three obesity indices, VAI had the strongest association with CKD compared to BMI and WC in women. In addition, VAI in women should be given more importance in the screening for CKD among the middle-aged and elderly Taiwanese population.

## Introduction

Obesity is associated with several metabolic disorders and might result in increased risks of adverse health outcomes. Globally, the proportion of adults with a body mass index (BMI) of 25 or above increased from 28.8% in 1980 to 36.9% in 2013 for men and from 29.8% to 38.0% for women. The increase was observed in both developed and developing countries. The proportion of adults with overweight and obesity in Taiwan is significantly higher than that in Japan, South Korea, and other neighboring Asian countries, especially in females (1). In Taiwan, the prevalence of overweight and obesity among adults was reported to be 44.1%, of which 50.8% were men and 36.9% were women, according to 2005–2008 data (2). On the other hand, a large Taiwanese cohort study found that the prevalence of chronic kidney disease (CKD) is 11.9% in adults and 37.2% among the elderly (3). As one of the countries with a rapidly aging population with an increasing prevalence of diabetes, hypertension (HTN), and subsequently CKD, Taiwan has the highest prevalence and incidence of end-stage renal disease in the world (4). According to the annual report by the Bureau of National Health Insurance in 2007, in Taiwan, patients with end-stage renal disease accounted for 0.23% of the local population but used 7.2% of the healthcare resources (5). Many risk factors were associated with the development or progression of CKD, such as diabetes, HTN, dyslipidemia, proteinuria, smoking, old age, heavy consumption of non-narcotic analgesics, and certain environmental and occupational exposures (6–11). Other than these well-known factors, many studies have demonstrated that obesity is also an important risk factor for CKD (12–15).

The relationship between obesity and CKD is complex and not yet fully understood. There is no consensus about which index is a better predictor for CKD. Previous studies showed BMI, as a representative of obesity, was associated with an increased risk of CKD (12–15). However, a prospective study from Korea showed that parameters of central obesity such as waist circumference (WC), waist-to-hip ratio, and waist-to-height ratio, but not BMI, are associated with a more rapid decline in renal function (16). Furthermore, a Japanese study showed that overweight and obesity were associated with an increased risk for CKD, and there was a gender difference in these associations (17).

Furthermore, a newer obesity index, the visceral adiposity index (VAI), was proposed by Amato and his research group in 2009. VAI is a novel sex-specific index based on WC, BMI, triglycerides, and high-density lipoprotein (HDL) and can estimate the visceral adiposity dysfunction associated with cardiometabolic risk (18). However, few studies have investigated the relationship between VAI and CKD, and the gender differences in this relationship. Also, there has not been relative research investigating about the association between obesity indices and CKD in the middle-aged and elderly population. Additionally, it is not known whether the VAI, which includes blood lipids, could outperform conventional indices in predicting CKD and whether there are gender differences in the association between obesity indices and CKD in the middle-aged and elderly Taiwanese population.

Therefore, this study aimed to investigate which of the three obesity indices has the strongest association with CKD and to explore whether there are gender differences in these relationships in the middle-aged and elderly Taiwanese population.

## Materials and Methods

### Study Design and Participants

**Figure 1:**
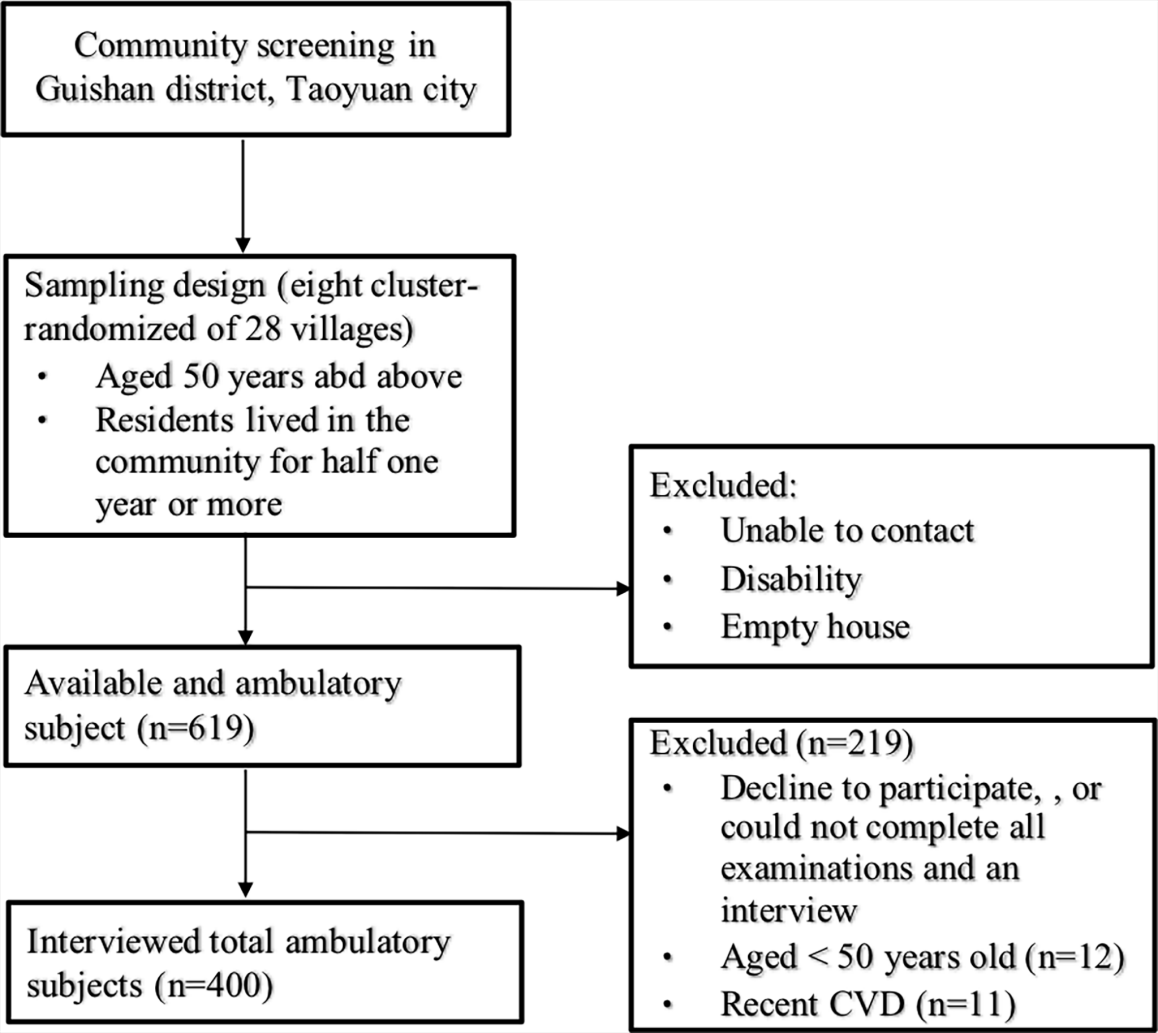
Flow chart of study subjects.

A community-based, cross-sectional study was conducted at the Linkou Chang Gung Memorial Hospital in Taoyuan City, Taiwan, between January and October 2014. We recruited 619 volunteers aged 50 years and above in a consecutive manner, from a community health promotion project in 2014, from 8 cluster-randomized of 28 villages in Guishan district, Taoyuan city. The inclusion criteria were: (1) subjects aged 50 years of age or above and (2) subjects residing in Guishan District for half one year or more. Participants were excluded if they (1) had functional disability, (2) declined to participate or could not complete all examinations and the interview, (3) had recent cardiovascular diseases (**Figure 1**). A total of 400 participants, including 141 men and 259 women, were eligible for the analysis. In addition, a sample size of 352 achieves 90% power using two tail(s), odds ratio (OR) = 2.0, probability of null hypothesis = 0.15, alpha error = 0.05, power = 0.9 and R^2^ for other confounding factors = 0.5 (19). A total of 400 subjects comprised the sample size of this study, which implied the sufficient statistical power. The study was approved by the Institutional Review Board of Chang Gung Memorial Hospital, and written informed consent was obtained from all participants before enrollment.

### Data Collection

Data collection comprised three parts: anthropometric measurements, laboratory tests, and structured questionnaires. For the anthropometric measurements, BMI, WC, blood pressure (BP), and heart rate were recorded. BMI was calculated as weight (kg) divided by height squared (m^2^). WC was measured at the mid-point between the lower border of the rib cage and the upper iliac crest on the mid-axillary line. Systolic BP (SBP), diastolic BP (DBP), and heart rate was checked at least twice after 5 minutes of rest on a chair. Laboratory tests included blood urea nitrogen (BUN), serum creatinine, fasting blood glucose, total cholesterol, high-density lipo-protein-cholesterol (HDL-C), low-density lipo-protein-cholesterol (LDL-C), TG, and urine albumin to creatinine ratio. Venous blood samples were collected after overnight fasting for at least 12 hours, and serum samples were used for biochemistry analysis. Urine specimens were obtained in the morning and scheduled to avoid the menstrual periods. A standardized biochemistry analyzer (Hitachi LST008, Hitachinaka shi, Japan) was used for determination of laboratory tests. Personal history was collected by a standard interview, and the information in the structured questionnaires included smoking/drinking habits, past medical history and current medication use. Obesity was defined as a BMI ≥ 27 kg/m^2^ or WC ≥ 90 cm for males and ≥ 80 cm for females, according to the criteria of the Department of Health in Taiwan. HTN was defined as SBP ≧ 140 mmHg or DBP ≧ 90 mmHg, current use of antihypertensive medications, or history of HTN. The definition of HTN was based on the 2015 guidelines of the Taiwan Society of Cardiology and the Taiwan Hypertension Society for the Management of Hypertension (20). Diabetes mellitus (DM) was defined as a fasting blood glucose ≥ 126 mg/dL, the use of oral antidiabetic drugs or insulin therapy, or history of DM. The definition of DM was based on the Diabetes Association of Republic of China (DAROC) clinical practice guidelines for diabetes care- 2018 (21). Hyperlipidemia was defined as LDL-C ≥ 130 mg/dL, TG ≥ 150 mg/dL, or total cholesterol ≥ 200 mg/dL, the use of lipid-lowering medication, or history of hyperlipidemia, according to the 2017 guidelines for management of dyslipidemia and prevention of cardiovascular disease by the American Association of Clinical Endocrinologists and American College of Endocrinology (22).

### Definition of VAI

VAI was calculated using the following formula (18):


$$\text{Men}:\;\text{VAI}=[\frac{\text{WC }\left(\text{cm}\right)}{39.68\;+\left(1.88\;\times \text{ BMI}\left(\frac{\text{kg}}{{\text{m}}^{2}}\right)\right)\;}]\times [\frac{\text{TG }\left(\text{mmol}/\text{l}\right)}{1.03}]\times [\frac{1.31}{HDL-C(mmol/l)}]$$

$$\text{Women}:\text{VAI}=[\frac{\text{WC}\;(cm)}{36.58+(1.89\;\times \;BMI\left(\frac{kg}{m^{2}}\right))}]\times [\frac{TG(mmol/l)}{0.81}]\times [\frac{1.52}{HDL-C(mmol/l)}]$$

### Definition of CKD

CKD is defined as decreased renal function with an estimated glomerular filtration rate (eGFR) <60 mL/min/1.73m^2^ or the presence of albuminuria (urine albumin to creatinine ratio ≥30 mg/g), according to the 2005 guidelines of Kidney Disease: Improving Global Outcomes (KDIGO) (23). The eGFR was calculated using the modified equation of Modification of Diet in Renal Disease (MDRD) for Chinese CKD patients: eGFR (ml/min per 1.73 m^2^) = 175 × Creatinine (mg/dl)^-1.234^ × Age^-0.179^× 0.79 (for women) (24).

### Statistical Analysis

The sample size determination was based on the G*power 3.1 software. General characteristics were expressed as mean ± standard deviation (SD) and median with inter-quartile range (IQR) for continuous variables and number (%) for categorical variables. P-values were derived from Mann-Whitney U test for continuous variables and chi-square test for categorical variables. Multivariate logistic regression models were developed to investigate the independence of obesity indices associated with CKD. We used the area under the receiver-operating characteristic curve (AUC) and 95% confidence intervals (CI) to assess the discriminatory power of each obesity index to predict the risk for CKD. All statistical analyses were performed using SPSS version 22.0 software (SPSS Inc., Chicago, IL). A p-value <0.05 was considered significant.

## Results

### Clinical Characteristics of the Study Population According to Gender and CKD Status

A total of 400 participants aged 50 years and above were enrolled in this study. The general characteristics of the study population divided by gender are shown in **Table 1**. Among the total participants, 141 were men, and 81 (20.25%) patients in the total cohort had CKD. The average age of the subjects was 64.47 ± 8.45 years, and men were significantly older. Among the three obesity indices, WC was significantly larger in men, and VAI was significantly higher in women. Furthermore, there were significant differences in BUN, creatinine, eGFR, HDL-C, LDL-C, TC, and DBP. The mean eGFR of the total cohort was 112.97 ± 33.43 ml/min/1.73m^2^, while the median eGFR among men was significantly lower (p < 0.001). In addition, the proportion of subjects who were current smokers and consumed alcohol was significantly higher in men than in women. However, there was no significant difference between the two groups in terms of the past medical history, such as presence of HTN, DM, hyperlipidemia and CKD and current medication use including Chinese herb and analgesics use.

**Table 1:** General characteristics of the study population according to gender.

Variables	Total (n = 400) mean ± SD	Men (n = 141) median (IQR)	Women (n = 259) median (IQR)	p value
Age (year)	64.47 ± 8.45	64 [60, 72]	62 [57, 70]	0.005
BMI (kg/m^2^)	24.55 ± 3.57	24.6 [22.2, 27.2]	23.9 [22.4, 26.7]	0.32
Waist circumference (cm)	85.07 ± 9.68	89 [84, 95]	81 [77, 88]	<0.001
VAI	1.84 ± 1.38	1.4 [0.8, 2.1]	1.5 [1.0, 2.3]	0.046
BUN (mg/dL)	15.92 ± 5.99	15.7 [13.4, 9.4]	14.3 [11.8, 17.5]	0.003
Creatinine (mg/dL)	0.78 ± 0.43	0.9 [0.8, 1.0]	0.6 [0.6, 0.7]	<0.001
eGFR (ml/min/1.73m^2^)	112.97 ± 33.43	93.6 [83.0, 117.2]	123.9 [101.7, 137.3]	<0.001
Glucose(AC) (mg/dL)	96.23 ± 25.73	92 [86, 102]	90 [82, 100]	0.06
HDL-C (mg/dL)	54.43 ± 13.93	46 [39, 53]	57 [48, 66]	<0.001
LDL-C (mg/dL)	118.37 ± 32.11	109 [86, 132]	119 [101, 141]	0.002
T-Cholesteterol (mg/dL)	197.15 ± 35.71	183 [158, 209]	202 [179, 226]	<0.001
Triglyceride (mg/dL)	122.07 ± 65.97	110 [76, 151]	106 [79, 144]	0.82
SBP (mmHg)	129.50 ± 16.71	129 [119, 140]	128 [117, 139]	0.40
DBP (mmHg)	76.93 ± 11.36	80 [72, 86]	76 [69, 82]	0.002
Current smoking, n(%)	43 10.75%	35 24.8%	8 3.1%	<0.001
Alcohol drinking ≧2 times/week, n(%)	75 18.75%	47 33.3%	28 10.8%	<0.001
ACR ≧30 mg/g, n(%)	75 18.75%	29 20.6%	46 17.8%	0.49
HTN, n(%)	201 50.25%	77 54.6%	124 47.9%	0.20
DM, n(%)	79 19.75%	30 21.3%	49 18.9%	0.57
Hyperlipidemia, n(%)	260 65.00%	84 59.6%	176 68.0%	0.09
CKD, n(%)	81 20.25%	31 22.0%	50 19.3%	0.52
Chinese herb use, n(%)	129 32.25%	50 35.5%	79 30.5%	0.31
Analgesics use, n(%)	32 8.00%	11 7.8%	21 8.1%	0.91

Clinical characteristics are presented as mean ± standard deviation (SD) and median with inter-quartile range (IQR) for continuous variables and n (%) for categorical variables. P-value were derived from Mann-Whitney U test for continuous variables and chi-square test for categorical variables.

BMI, body mass index; VAI, visceral adiposity index; BUN, blood urea nitrogen; eGFR, estimated glomerular filtration rate; HDL-C, high-density lipoprotein cholesterol; LDL-C, low-density lipoprotein cholesterol; T-cholesterol, total cholesterol; SBP, systolic blood pressure; DBP, diastolic blood pressure; ACR, urine albumin creatinine ratio; HTN, hypertension; DM, diabetes mellitus; CKD, chronic kidney disease.

**Table 2** shows the general characteristics by gender in the study population with CKD and those with no CKD. In men, there was no significant difference between the CKD and non-CKD groups among the three obesity indices. However, in women, only VAI was significantly higher in subjects with CKD (1.9 [1.1, 3.4]) than in subjects without CKD (1.5 [1.0, 2.2]) (p-value = 0.03). Furthermore, in men, significant differences were found in BUN, creatinine, eGFR, glucose AC, HDL-C, and SBP. In women, significant differences were found in creatinine, eGFR, LDL-C, TG, SBP, and DBP. Unexpectedly, in both men and women, the proportion of those who were current smokers and alcohol consumers was lower in the CKD group than in the non-CKD group. This might be because they quit smoking and alcohol consumption after being diagnosed with CKD. About the past medical history, the prevalence of HTN and DM but not hyperlipidemia was significantly higher in the CKD group than in the non-CKD group, in both men and women. About the current medication use, only the prevalence of Chinese herb use was significantly higher in the non-CKD group than in the CKD group, only in men.

**Table 2:** General characteristics according to gender in the study population with CKD and non-CKD.

Variables	Men			Women		
	non CKD (n = 110) median (IQR)	CKD (n = 31) median (IQR)	p value	non CKD (n = 209) median (IQR)	CKD (n = 50) median (IQR)	p value
Age (year)	64 [60, 70]	66 [60, 78]	0.18	62 [57, 69]	64 [58, 73]	0.13
BMI (kg/m^2^)	24.1 [22.1, 26.9]	25.2 [22.5, 28.1]	0.23	23.9 [22.3, 26.5]	24.8 [22.6, 27.2]	0.24
Waist circumference (cm)	88 [83, 94]	91 [86, 100]	0.05	81 [77, 87]	84 [77, 89]	0.31
VAI	1.4 [0.8, 1.9]	1.7 [0.9, 3.4]	0.05	1.5 [1.0, 2.2]	1.9 [1.1, 3.4]	0.03
BUN (mg/dL)	15.2 [13.1, 18.2]	17.9 [14.2, 23.9]	0.01	14.4 [11.9, 17.4]	14.1 [11.0, 20.7]	0.90
Creatinine (mg/dL)	0.9 [0.8, 1.0]	1.1 [0.8, 1.5]	<0.001	0.6 [0.6, 0.7]	0.7 [0.6, 0.9]	0.01
eGFR (ml/min/1.73m^2^)	98.2 [85.0, 118.4]	68.7 [51.0, 105.2]	<0.001	126.3 [106.0, 138.1]	109.7 [76.5, 136.8]	0.01
Glucose(AC) (mg/dL)	91 [86, 99]	98 [85, 129]	0.04	89.0 [82.0, 97.0]	92.5 [84.0, 108.8]	0.08
HDL-C (mg/dL)	47 [41, 55]	42 [36, 48]	0.02	58.0 [49.5, 66.0]	53.5 [44.8, 62.8]	0.05
LDL-C (mg/dL)	111 [87, 134]	105 [83, 130]	0.42	121 [102, 143]	110 [95, 129]	0.02
T-Cholesteterol (mg/dL)	183 [163, 212]	184 [149, 206]	0.39	204 [183, 228]	199 [176, 217]	0.13
Triglyceride (mg/dL)	110 [73, 144]	107 [91, 186]	0.22	104 [77, 138]	120 [85, 184]	0.01
SBP (mmHg)	128 [117, 139]	137 [127, 150]	0.003	127 [117, 138]	135 [121, 144]	0.02
DBP (mmHg)	80 [71, 85]	82 [74, 87]	0.38	75 [68, 82]	79 [71, 85]	0.02
Current smoking, n(%)	28 25.5%	7 22.6%	0.74	7 3.3%	1 2.0%	0.62
Alcohol drinking ≧2 times/week, n(%)	44 40.0%	3 9.7%	0.002	24 11.5%	4 8.0%	0.48
ACR ≧30 mg/g, n(%)	0 0.0%	29 93.5%	<0.001	0 0.0%	46 92.0%	<0.001
HTN, n(%)	53 48.2%	24 77.4%	0.004	89 42.6%	35 70.0%	<0.001
DM, n(%)	15 13.6%	15 48.4%	<0.001	34 16.3%	15 30.0%	0.03
Hyperlipidemia, n(%)	62 58.2%	20 64.5%	0.53	139 66.5%	37 74.0%	0.31
Chinese herb use, n(%)	44 40.0%	6 19.4%	0.03	67 32.1%	12 24.0%	0.27
Analgesics use, n(%)	7 6.4%	4 12.9%	0.23	193 92.3%	45 90.0%	0.59

Clinical characteristics are presented as median with inter-quartile range (IQR) for continuous variables and n (%) for categorical variables. P-value were derived from Mann-Whitney U test for continuous variables and chi-square test for categorical variables.

BMI, body mass index; VAI, visceral adiposity index; BUN, blood urea nitrogen; eGFR, estimated glomerular filtration rate; HDL-C, high-density lipoprotein cholesterol; LDL-C, low-density lipoprotein cholesterol; T-cholesterol, total cholesterol; SBP, systolic blood pressure; DBP, diastolic blood pressure; ACR, urine albumin creatinine ratio; HTN, hypertension; DM, diabetes mellitus; CKD, chronic kidney disease.

### Associations Between the Obesity Indices and Chronic Kidney Disease According to Gender

Multivariate logistic regression analysis of the three obesity indices associated with the risk of CKD in men and women is shown in **Table 3**. Model 1 was unadjusted; Model 2 was adjusted for age; Model 3 was adjusted for age, smoking habits, past medical history including DM, HTN and hyperlipidemia, Chinese herb and analgesics use. After adjusting for the above-mentioned confounding factors, VAI in women (OR: 1.32, 95% CI: 1.04-1.69, p = 0.02) was still significantly associated with CKD; however, this was not seen in men (OR: 1.20, 95% CI: 0.85-1.69, p = 0.30) (**Table 3**-model 3).

**Table 3:** Multivariate logistic regression analysis of different obesity indices associated with the risk of CKD in men and women.

Variables		Model 1			Model 2			Model 3		
		OR	95% CI	P value	OR	95% CI	P value	OR	95% CI	P value
Men (n=141)										
VAI		1.35	(1.04-1.75)	0.02	1.37	(1.04-1.79)	0.02	1.20	(0.85-1.69)	0.30
BMI (kg/m^2^)		1.07	(0.97-1.19)	0.17	1.11	(0.99-1.23)	0.06	1.08	(0.95-1.22)	0.26
Waist circumference (cm)		1.04	(1.00-1.08)	0.048	1.05	(1.01-1.09)	0.03	1.03	(0.98-1.08)	0.21
Women (n=259)										
VAI		1.44	(1.17-1.78)	0.001	1.43	(1.16-1.76)	0.001	1.32	(1.04-1.69)	0.02
BMI (kg/m^2^)		1.04	(0.95-1.14)	0.41	1.04	(0.95-1.13)	0.45	0.99	(0.90-1.09)	0.82
Waist circumference (cm)		1.02	(0.98-1.05)	0.37	1.01	(0.98-1.05)	0.54	0.99	(0.95-1.03)	0.65

Model 1: unadjusted.

Model 2: adjusted for age.

Model 3: adjusted for factors in model 2 plus smoking, DM, HTN, hyperlipidemia, Chinese herb use, analgesics use.

**Figure 2:**
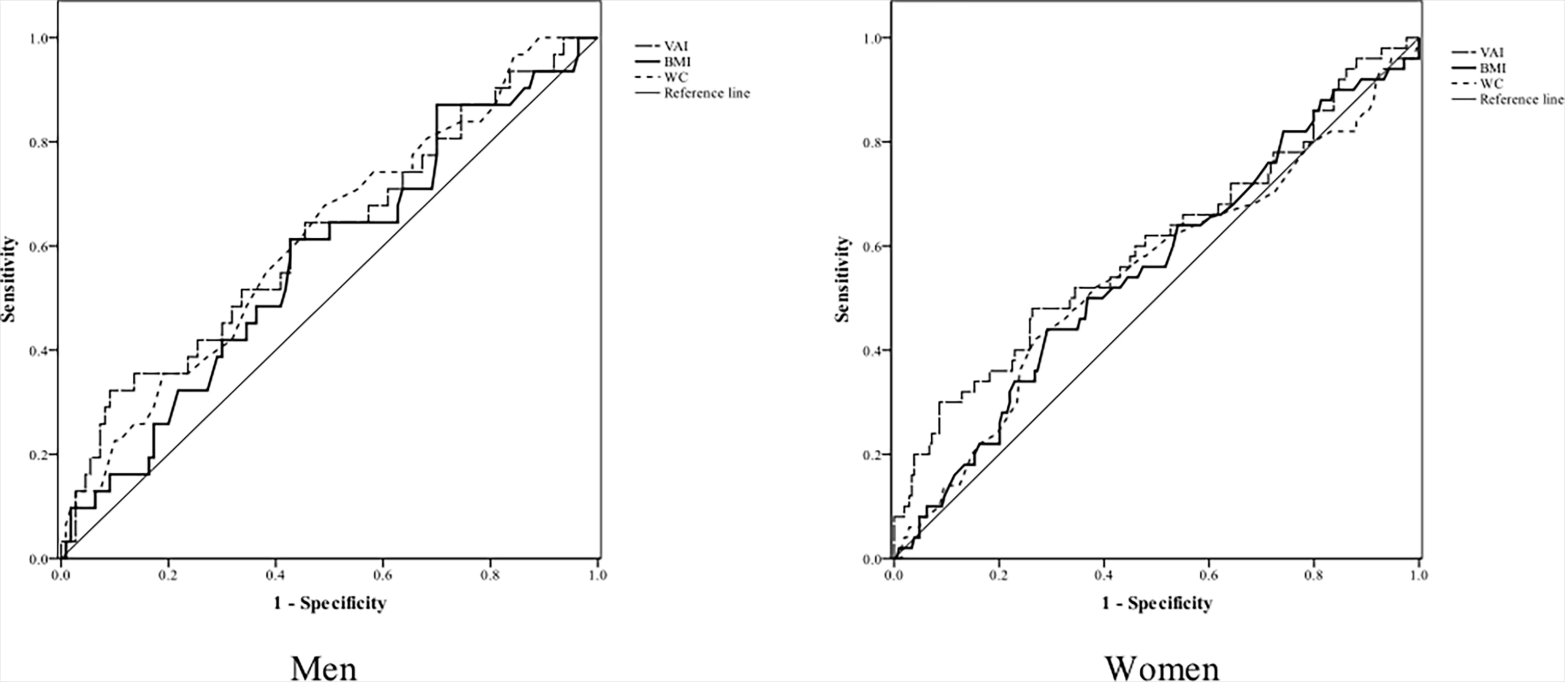
ROC curves for obesity indices as discriminators of CKD in men and women.

**Table 4** and **Figure 2** show the AUC scores (and 95% CIs), sensitivity, and specificity by the optimized cut-off points for the three obesity indices in predicting CKD according to gender. Only VAI was significantly capable of predicting CKD in women but not in men, and all three obesity indices had no statistically significant ability to predict CKD in men.

**Table 4:** The areas under ROC curve (AUC), sensitivity, and specificity by the optimized cut-off points for obesity indices in predicting CKD according to gender.

Variables		AUC (95% CI)		p value	Cut-off point	Sensitivity	Specificity
Men (n=141)							
VAI		0.61	(0.50-0.73)	0.05	2.86	0.32	0.91
BMI (kg/m^2^)		0.57	(0.46-0.69)	0.23	24.95	0.61	0.57
Waist circumference (cm)		0.61	(0.50-0.73)	0.05	88.50	0.68	0.51
Women (n=259)							
VAI		0.60	(0.51-0.70)	0.03	2.03	0.48	0.74
BMI (kg/m^2^)		0.55	(0.46-0.64)	0.24	25.75	0.44	0.71
Waist circumference (cm)		0.55	(0.45-0.64)	0.31	86.50	0.42	0.73

## Discussion

The main finding of this cross-sectional study was that among three different obesity indices, only VAI was significantly associated with CKD in the middle-aged and elderly female Taiwanese population. Few studies have compared the association between obesity indices and CKD, and the gender differences in this relationship. And, the predictive power of traditional measurements for chronic diseases is still debatable. To our knowledge, our study has found for the first time that among the middle-aged and elderly population, VAI had a better capability for predicting CKD compared to the traditional indices, only in women, and BMI and WC did not effectively identify the risk of CKD in both genders.

Obesity is closely associated with HTN, DM, and hyperlipidemia, all of which are unfavorable factors to renal function (8, 11, 25). Although the prevalence of HTN and DM was higher in the CKD group in our study, VAI was still significantly associated with CKD in women after adjusting these factors with known impacts on renal function. VAI was identified as an index that could be used as a surrogate marker of “adipose tissue dysfunction.” Further, VAI had higher sensitivity and specificity than conventional parameters such as WC and BMI and could significantly improve the risk assessment of cardiovascular diseases associated with obesity (18). Meanwhile, obesity has been recently shown to be a risk factor for CKD, which is independent of the other risks mentioned above (26, 27). The possible mechanisms of this obesity-induced renal injury are adipocyte-derived bioactive factors and triggering a low-grade chronic inflammatory state. Fat distribution, specifically visceral adiposity, is a key determinant of renal dysfunction, even in individuals with normal weight (26).

Many methods can be used to assess visceral adiposity. Electronic methods used as the gold standard, include, densitometry (dual-energy X-ray absorptiometry), magnetic resonance imaging, and computed tomography, which provide the assessment of body and visceral fat with high precision (28). These methods are technically complex, and too expensive and time-consuming to be used routinely in clinical practice. VAI has the advantages of low cost, simple measurement, and good social profit. We suggest that VAI as an effective marker of visceral obesity is the simpler and more significant predictor of CKD than the traditional indices in women.

An interesting finding of our study was a gender difference in the association between CKD and obesity indices. A significant association between CKD and VAI was seen only in women by multiple logistic regression and AUC analyses. A similar finding was presented by Dai et al., who reported that VAI and lipid accumulation product index was superior to BMI, WC, and waist-to-height ratio for predicting CKD only in women in the Northeast Chinese rural population (29). The reason for the insignificant association between obesity indices and CKD in men is unclear. One possible reason for the sex difference in the association between obesity indices and CKD is that adipose tissue function and deposition differ by sex. Palmer BF conducted a review showing that since estrogens could facilitate adipose tissue deposition and function, men and postmenopausal women tend to accrue more visceral fat, leading to the classic android body shape, which has been highly correlated to increased cardiovascular risk; whereas women accrue more fat in the subcutaneous depot prior to menopause, a feature which offers protection from the negative consequences associated with obesity and the metabolic syndrome (30). In our study, the mean age of female participants was 63.55 ± 8.23 years, which is the age of the postmenopausal period. Another possible reason is that sex hormones affect the kidneys and influence the progression of renal disease. Estradiol suppresses total collagen synthesis by the mesangial cells, whereas testosterone does not affect collagen synthesis, which might explain the slower development of glomerulosclerosis in women, and therefore the protective effect on the progression of renal disease in females (31). These findings suggest that sex could act as a renal disease modifier.

Our study had several limitations. First, the prevalence of CKD in our study was higher than that reported in a Taiwanese national sample survey (11.93%) (3), and participants were enrolled from a community in northern Taiwan, which may be due to the possible Neyman bias and raises uncertainty of the external validity of the findings. Therefore, the study was not representative of the entire country. Second, the results might be confounded by unmeasured factors, such as structural or functional kidney abnormality, and certain environmental or occupational exposures. Lastly, our findings are based on data originating from a cross-sectional study, which limits our ability to infer a causal relationship between obesity indices and CKD, and further exploration is needed.

In conclusion, our analysis found that among the three obesity indices, VAI was significantly associated with the risk of CKD in women after adjusting for confounding factors. In addition, we also found that VAI showed the strongest ability for predicting CKD compared to BMI and WC only in women.

## Data Availability Statement

The original contributions presented in the study are included in the article/supplementary material. Further inquiries can be directed to the corresponding author.

## Ethics Statement

The studies involving human participants were reviewed and approved by Institutional Review Board of Chang-Gung Medical Foundation. The patients/participants provided their written informed consent to participate in this study. Written informed consent was obtained from the individual(s) for the publication of any potentially identifiable images or data included in this article.

## Author Contributions

J-YC planned and conceptualized the work. Data collection and analysis was performed by J-YC. I-JC wrote the original draft, and J-YC revised the draft. J-YC, L-TH, M-CL, Y-JC, and M-TT supervised the work. All authors contributed to the article and approved the submitted version.

## Funding

This study was supported by Chang Gung Memorial Hospital (grants CORPG3C0171~3C0172, CZRPG3C0053, CORPG3G0021, CORPG3G0022, CORPG3G0023).

## Conflict of Interest

The authors declare that the research was conducted in the absence of any commercial or financial relationships that could be construed as a potential conflict of interest.

## Publisher’s Note

All claims expressed in this article are solely those of the authors and do not necessarily represent those of their affiliated organizations, or those of the publisher, the editors and the reviewers. Any product that may be evaluated in this article, or claim that may be made by its manufacturer, is not guaranteed or endorsed by the publisher.
